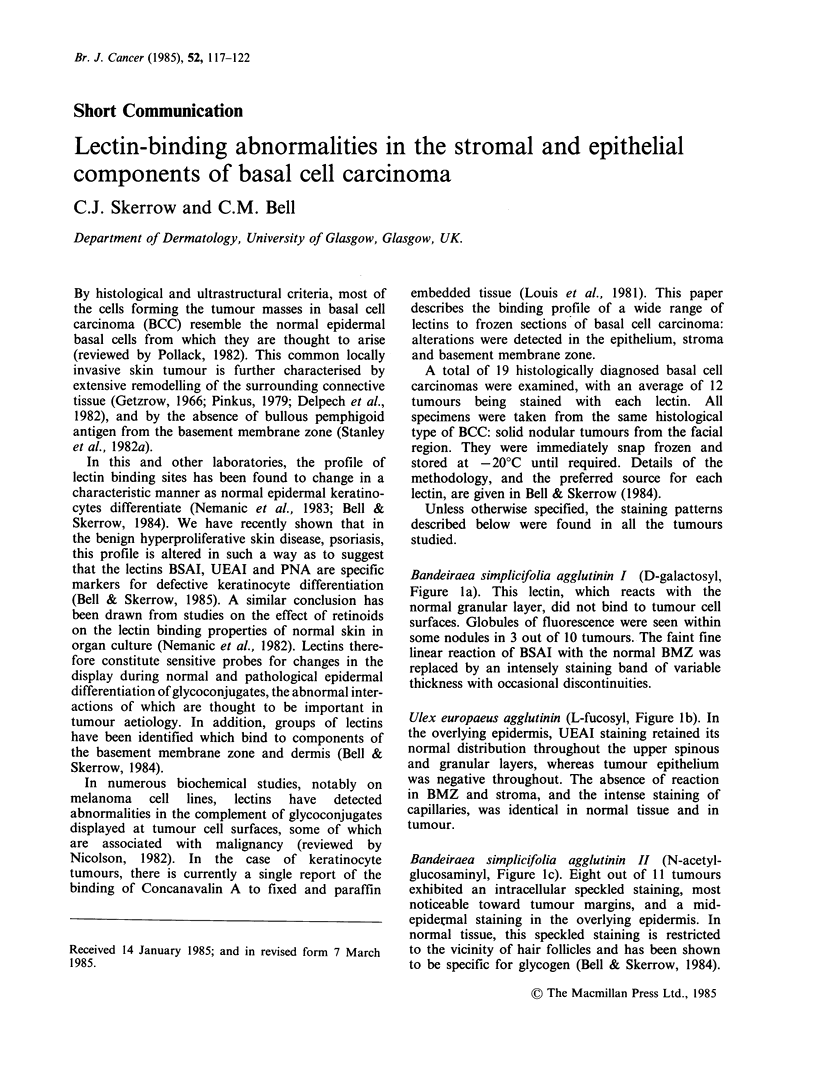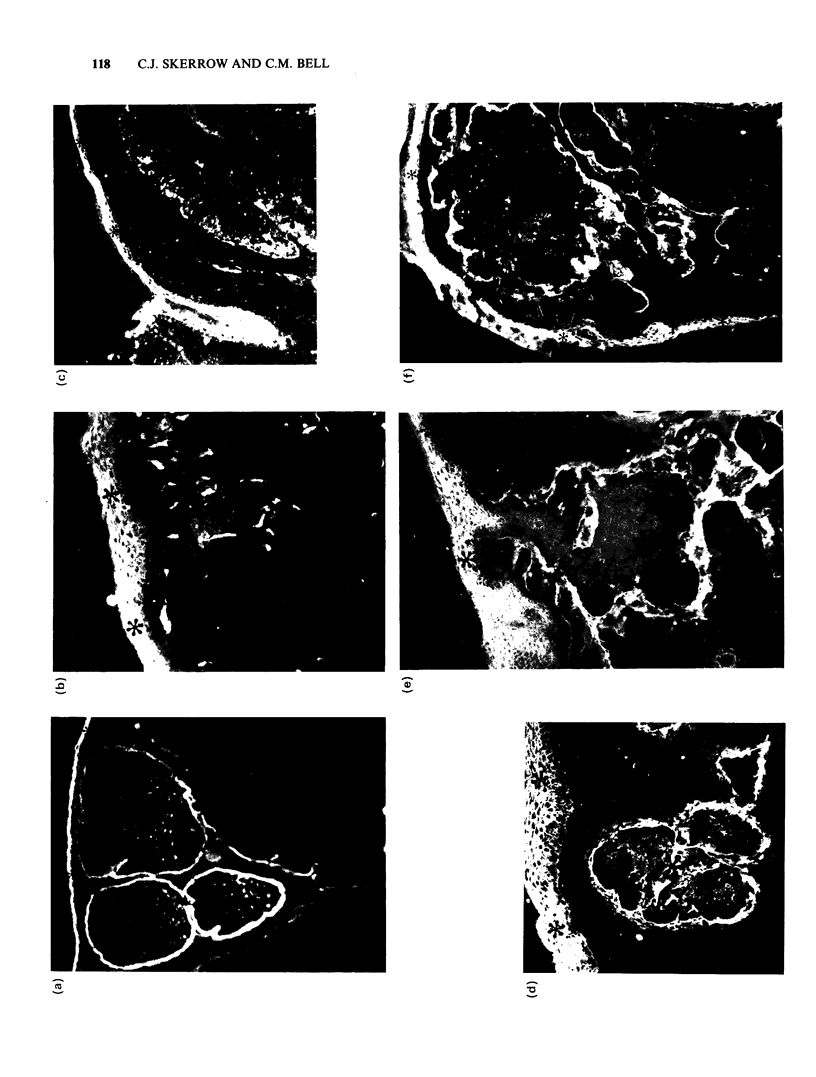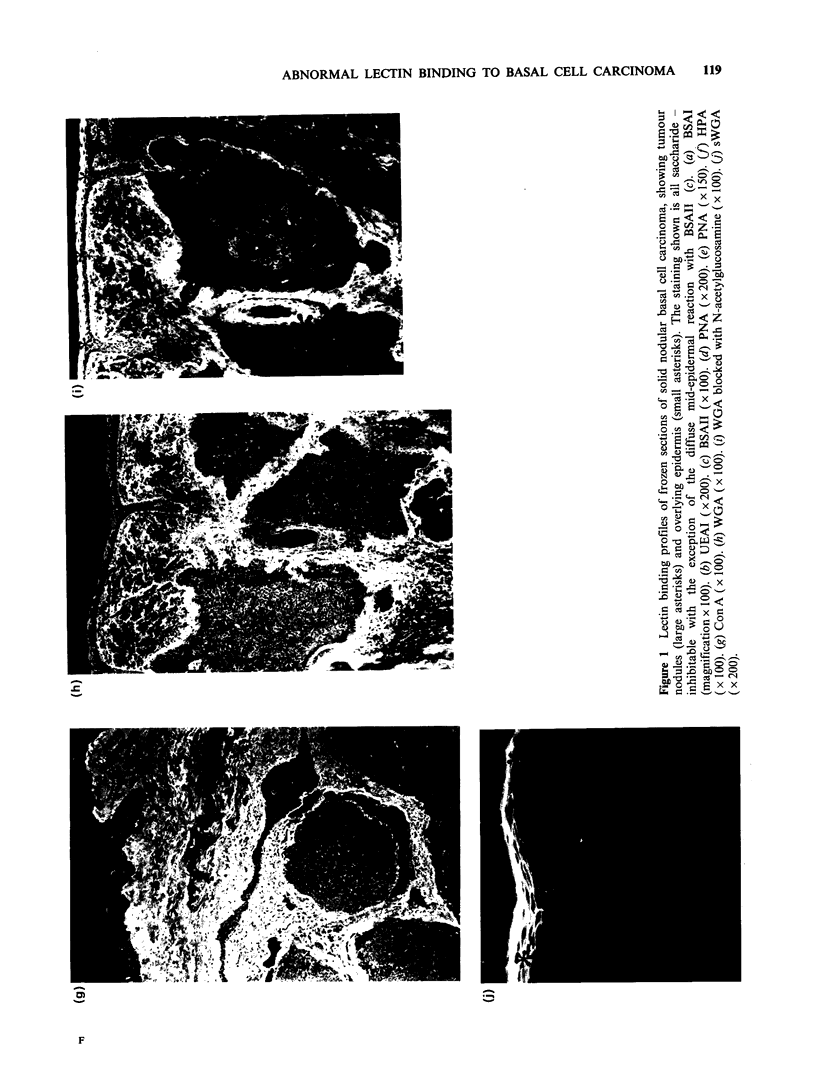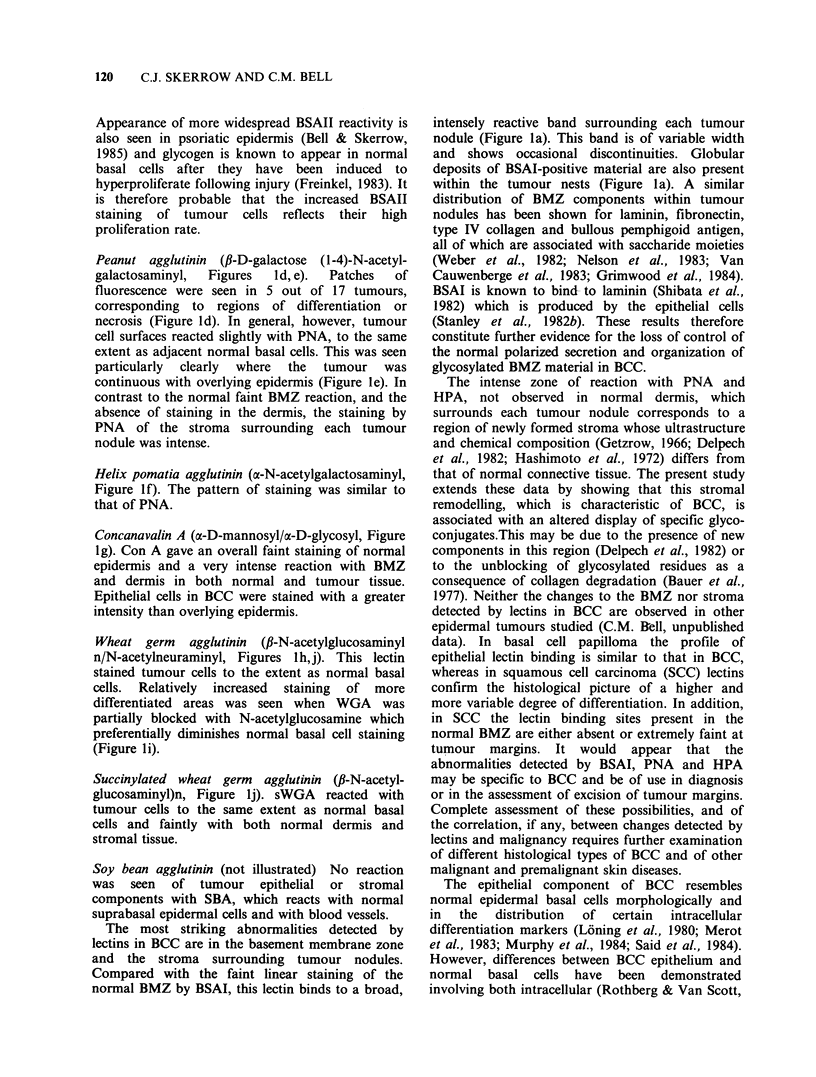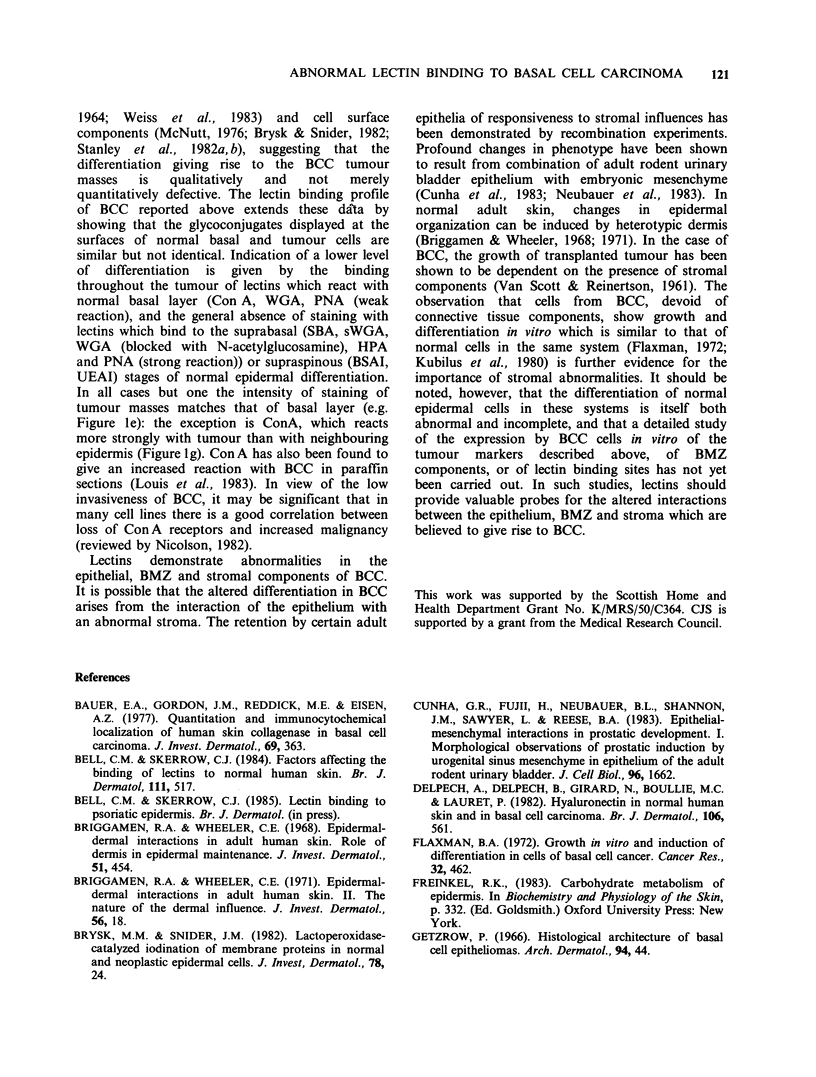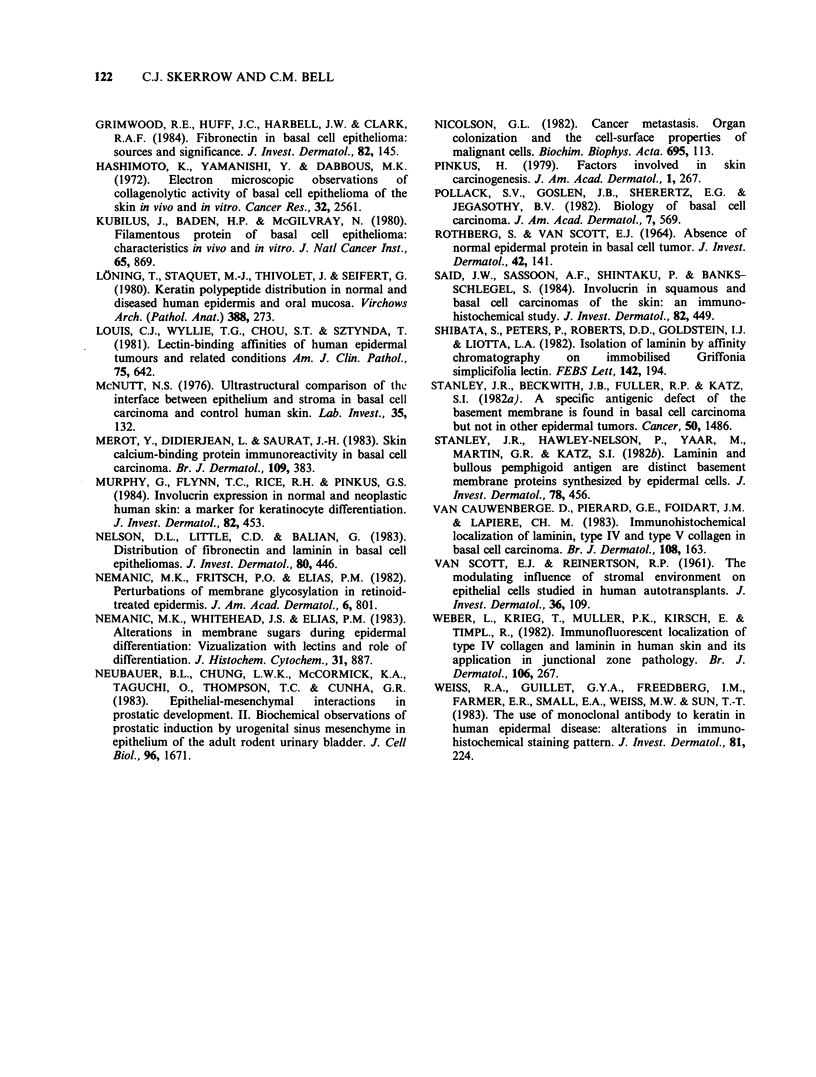# Lectin-binding abnormalities in the stromal and epithelial components of basal cell carcinoma.

**DOI:** 10.1038/bjc.1985.158

**Published:** 1985-07

**Authors:** C. J. Skerrow, C. M. Bell

## Abstract

**Images:**


*Br. J. Cancer* (1985), **52**, 117–122

### Short Communication

### Lectin-binding abnormalities in the stromal and epithelial components of basal cell carcinoma

C.J. Skerrow and C.M. Bell

*Department of Dermatology, University of Glasgow, Glasgow, UK.*

By histological and ultrastructural criteria, most of the cells forming the tumour masses in basal cell carcinoma (BCC) resemble the normal epidermal basal cells from which they are thought to arise (reviewed by Pollack, 1982). This common locally invasive skin tumour is further characterised by extensive remodelling of the surrounding connective tissue (Getzrow, 1966; Pinkus, 1979; Delpech *et al.*, 1982), and by the absence of bullous pemphigoid antigen from the basement membrane zone (Stanley *et al.*, 1982*a*).

In this and other laboratories, the profile of lectin binding sites has been found to change in a characteristic manner as normal epidermal keratinocytes differentiate (Nemanic *et al.*, 1983; Bell & Skerrow, 1984). We have recently shown that in the benign hyperproliferative skin disease, psoriasis, this profile is altered in such a way as to suggest that the lectins BSAI, UEAI and PNA are specific markers for defective keratinocyte differentiation (Bell & Skerrow, 1985). A similar conclusion has been drawn from studies on the effect of retinoids on the lectin binding properties of normal skin in organ culture (Nemanic *et al.*, 1982). Lectins therefore constitute sensitive probes for changes in the display during normal and pathological epidermal differentiation of glycoconjugates, the abnormal interactions of which are thought to be important in tumour aetiology. In addition, groups of lectins have been identified which bind to components of the basement membrane zone and dermis (Bell & Skerrow, 1984).

In numerous biochemical studies, notably on melanoma cell lines, lectins have detected abnormalities in the complement of glycoconjugates displayed at tumour cell surfaces, some of which are associated with malignancy (reviewed by Nicolson, 1982). In the case of keratinocyte tumours, there is currently a single report of the binding of Concanavalin A to fixed and paraffin embedded tissue (Louis *et al.*, 1981). This paper describes the binding profile of a wide range of lectins to frozen sections of basal cell carcinoma: alterations were detected in the epithelium, stroma and basement membrane zone.

A total of 19 histologically diagnosed basal cell carcinomas were examined, with an average of 12 tumours being stained with each lectin. All specimens were taken from the same histological type of BCC: solid nodular tumours from the facial region. They were immediately snap frozen and stored at −20°C until required. Details of the methodology, and the preferred source for each lectin, are given in Bell & Skerrow (1984).

Unless otherwise specified, the staining patterns described below were found in all the tumours studied.

*Bandeiraea simplicifolia agglutinin I* (D-galactosyl, Figure 1a). This lectin, which reacts with the normal granular layer, did not bind to tumour cell surfaces. Globules of fluorescence were seen within some nodules in 3 out of 10 tumours. The faint fine linear reaction of BSAI with the normal BMZ was replaced by an intensely staining band of variable thickness with occasional discontinuities.

*Ulex europaeus agglutinin* (L-fucosyl, Figure 1b). In the overlying epidermis, UEAI staining retained its normal distribution throughout the upper spinous and granular layers, whereas tumour epithelium was negative throughout. The absence of reaction in BMZ and stroma, and the intense staining of capillaries, was identical in normal tissue and in tumour.

*Bandeiraea simplicifolia agglutinin II* (N-acetyl-glucosaminyl, Figure 1c). Eight out of 11 tumours exhibited an intracellular speckled staining, most noticeable toward tumour margins, and a mid-epidermal staining in the overlying epidermis. In normal tissue, this speckled staining is restricted to the vicinity of hair follicles and has been shown to be specific for glycogen (Bell & Skerrow, 1984).

Received 14 January 1985; and in revised form 7 March 1985.

© The Macmillan Press Ltd., 1985

Appearance of more widespread BSAII reactivity is also seen in psoriatic epidermis (Bell & Skerrow, 1985) and glycogen is known to appear in normal basal cells after they have been induced to hyperproliferate following injury (Freinkel, 1983). It is therefore probable that the increased BSAII staining of tumour cells reflects their high proliferation rate.

*Peanut agglutinin* (β-D-galactose (1-4)-N-acetyl-galactosaminyl, Figures 1d, e). Patches of fluorescence were seen in 5 out of 17 tumours, corresponding to regions of differentiation or necrosis (Figure 1d). In general, however, tumour cell surfaces reacted slightly with PNA, to the same extent as adjacent normal basal cells. This was seen particularly clearly where the tumour was continuous with overlying epidermis (Figure 1e). In contrast to the normal faint BMZ reaction, and the absence of staining in the dermis, the staining by PNA of the stroma surrounding each tumour nodule was intense.

*Helix pomatia agglutinin* (α-N-acetylgalactosaminyl, Figure 1f). The pattern of staining was similar to that of PNA.

*Concanavalin A* (α-D-mannosyl/α-D-glycosyl, Figure 1g). Con A gave an overall faint staining of normal epidermis and a very intense reaction with BMZ and dermis in both normal and tumour tissue. Epithelial cells in BCC were stained with a greater intensity than overlying epidermis.

*Wheat germ agglutinin* (β-N-acetylglucosaminyl n/N-acetylneuraminyl, Figures 1h, j). This lectin stained tumour cells to the extent as normal basal cells. Relatively increased staining of more differentiated areas was seen when WGA was partially blocked with N-acetylglucosamine which preferentially diminishes normal basal cell staining (Figure 1i).

*Succinylated wheat germ agglutinin* (β-N-acetyl-glucosaminyl)n, Figure 1j). sWGA reacted with tumour cells to the same extent as normal basal cells and faintly with both normal dermis and stromal tissue.

*Soy bean agglutinin* (not illustrated) No reaction was seen of tumour epithelial or stromal components with SBA, which reacts with normal suprabasal epidermal cells and with blood vessels.

The most striking abnormalities detected by lectins in BCC are in the basement membrane zone and the stroma surrounding tumour nodules. Compared with the faint linear staining of the normal BMZ by BSAI, this lectin binds to a broad, intensely reactive band surrounding each tumour nodule (Figure 1a). This band is of variable width and shows occasional discontinuities. Globular deposits of BSAI-positive material are also present within the tumour nests (Figure 1a). A similar distribution of BMZ components within tumour nodules has been shown for laminin, fibronectin, type IV collagen and bullous pemphigoid antigen, all of which are associated with saccharide moieties (Weber *et al.*, 1982; Nelson *et al.*, 1983; Van Cauwenberge *et al.*, 1983; Grimwood *et al.*, 1984). BSAI is known to bind to laminin (Shibata *et al.*, 1982) which is produced by the epithelial cells (Stanley *et al.*, 1982*b*). These results therefore constitute further evidence for the loss of control of the normal polarized secretion and organization of glycosylated BMZ material in BCC.

The intense zone of reaction with PNA and HPA, not observed in normal dermis, which surrounds each tumour nodule corresponds to a region of newly formed stroma whose ultrastructure and chemical composition (Getzrow, 1966; Delpech *et al.*, 1982; Hashimoto *et al.*, 1972) differs from that of normal connective tissue. The present study extends these data by showing that this stromal remodelling, which is characteristic of BCC, is associated with an altered display of specific glyco-conjugates.This may be due to the presence of new components in this region (Delpech *et al.*, 1982) or to the unblocking of glycosylated residues as a consequence of collagen degradation (Bauer *et al.*, 1977). Neither the changes to the BMZ nor stroma detected by lectins in BCC are observed in other epidermal tumours studied (C.M. Bell, unpublished data). In basal cell papilloma the profile of epithelial lectin binding is similar to that in BCC, whereas in squamous cell carcinoma (SCC) lectins confirm the histological picture of a higher and more variable degree of differentiation. In addition, in SCC the lectin binding sites present in the normal BMZ are either absent or extremely faint at tumour margins. It would appear that the abnormalities detected by BSAI, PNA and HPA may be specific to BCC and be of use in diagnosis or in the assessment of excision of tumour margins. Complete assessment of these possibilities, and of the correlation, if any, between changes detected by lectins and malignancy requires further examination of different histological types of BCC and of other malignant and premalignant skin diseases.

The epithelial component of BCC resembles normal epidermal basal cells morphologically and in the distribution of certain intracellular differentiation markers (Löning *et al.*, 1980; Merot *et al.*, 1983; Murphy *et al.*, 1984; Said *et al.*, 1984). However, differences between BCC epithelium and normal basal cells have been demonstrated involving both intracellular (Rothberg & Van Scott,

1964; Weiss *et al.*, 1983) and cell surface components (McNutt, 1976; Brysk & Snider, 1982; Stanley *et al.*, 1982*a*, *b*), suggesting that the differentiation giving rise to the BCC tumour masses is qualitatively and not merely quantitatively defective. The lectin binding profile of BCC reported above extends these data by showing that the glycoconjugates displayed at the surfaces of normal basal and tumour cells are similar but not identical. Indication of a lower level of differentiation is given by the binding throughout the tumour of lectins which react with normal basal layer (Con A, WGA, PNA (weak reaction), and the general absence of staining with lectins which bind to the suprabasal (SBA, sWGA, WGA (blocked with N-acetylglucosamine), HPA and PNA (strong reaction)) or supraspinous (BSAI, UEAI) stages of normal epidermal differentiation. In all cases but one the intensity of staining of tumour masses matches that of basal layer (e.g. Figure 1e): the exception is ConA, which reacts more strongly with tumour than with neighbouring epidermis (Figure 1g). Con A has also been found to give an increased reaction with BCC in paraffin sections (Louis *et al.*, 1983). In view of the low invasiveness of BCC, it may be significant that in many cell lines there is a good correlation between loss of Con A receptors and increased malignancy (reviewed by Nicolson, 1982).

Lectins demonstrate abnormalities in the epithelial, BMZ and stromal components of BCC. It is possible that the altered differentiation in BCC arises from the interaction of the epithelium with an abnormal stroma. The retention by certain adult epithelia of responsiveness to stromal influences has been demonstrated by recombination experiments. Profound changes in phenotype have been shown to result from combination of adult rodent urinary bladder epithelium with embryonic mesenchyme (Cunha *et al.*, 1983; Neubauer *et al.*, 1983). In normal adult skin, changes in epidermal organization can be induced by heterotypic dermis (Briggamen & Wheeler, 1968; 1971). In the case of BCC, the growth of transplanted tumour has been shown to be dependent on the presence of stromal components (Van Scott & Reinertson, 1961). The observation that cells from BCC, devoid of connective tissue components, show growth and differentiation *in vitro* which is similar to that of normal cells in the same system (Flaxman, 1972; Kubilus *et al.*, 1980) is further evidence for the importance of stromal abnormalities. It should be noted, however, that the differentiation of normal epidermal cells in these systems is itself both abnormal and incomplete, and that a detailed study of the expression by BCC cells *in vitro* of the tumour markers described above, of BMZ components, or of lectin binding sites has not yet been carried out. In such studies, lectins should provide valuable probes for the altered interactions between the epithelium, BMZ and stroma which are believed to give rise to BCC.

This work was supported by the Scottish Home and Health Department Grant No. K/MRS/50/C364. CJS is supported by a grant from the Medical Research Council.

### References

BAUER, E.A., GORDON, J.M., REDDICK, M.E. & EISEN, A.Z. (1977). Quantitation and immunocytochemical localization of human skin collagenase in basal cell carcinoma. *J. Invest. Dermatol.*, **69**, 363.

BELL, C.M. & SKERROW, C.J. (1984). Factors affecting the binding of lectins to normal human skin. *Br. J. Dermatol*, **111**, 517.

BELL, C.M. & SKERROW, C.J. (1985). Lectin binding to psoriatic epidermis. *Br. J. Dermatol.* (in press).

BRIGGAMEN, R.A. & WHEELER, C.E. (1968). Epidermal-dermal interactions in adult human skin. Role of dermis in epidermal maintenance. *J. Invest. Dermatol.*, **51**, 454.

BRIGGAMEN, R.A. & WHEELER, C.E. (1971). Epidermal-dermal interactions in adult human skin. II. The nature of the dermal influence. *J. Invest. Dermatol.*, **56**, 18.

BRYSK, M.M. & SNIDER, J.M. (1982). Lactoperoxidase-catalyzed iodination of membrane proteins in normal and neoplastic epidermal cells. *J. Invest, Dermatol.*, **78**, 24.

CUNHA, G.R., FUJII, H., NEUBAUER, B.L., SHANNON, J.M., SAWYER, L. & REESE, B.A. (1983). Epithelial-mesenchymal interactions in prostatic development. I. Morphological observations of prostatic induction by urogenital sinus mesenchyme in epithelium of the adult rodent urinary bladder. *J. Cell Biol.*, **96**, 1662.

DELPECH, A., DELPECH, B., GIRARD, N., BOULLIE, M.C. & LAURET, P. (1982). Hyaluronectin in normal human skin and in basal cell carcinoma. *Br. J. Dermatol.*, **106**, 561.

FLAXMAN, B.A. (1972). Growth *in vitro* and induction of differentiation in cells of basal cell cancer. *Cancer Res.*, **32**, 462.

FREINKEL, R.K., (1983). Carbohydrate metabolism of epidermis. In *Biochemistry and Physiology of the Skin*, p. 332. (Ed. Goldsmith.) Oxford University Press: New York.

GETZROW, P. (1966). Histological architecture of basal cell epitheliomas. *Arch. Dermatol.*, **94**, 44.

GRIMWOOD, R.E., HUFF, J.C., HARBELL, J.W. & CLARK, R.A.F. (1984). Fibronectin in basal cell epithelioma: sources and significance. *J. Invest. Dermatol.*, **82**, 145.

HASHIMOTO, K., YAMANISHI, Y. & DABBOUS, M.K. (1972). Electron microscopic observations of collagenolytic activity of basal cell epithelioma of the skin *in vivo* and *in vitro*. *Cancer Res.*, **32**, 2561.

KUBILUS, J., BADEN, H.P. & McGILVRAY, N. (1980). Filamentous protein of basal cell epithelioma: characteristics *in vivo* and *in vitro*. *J. Natl Cancer Inst.*, **65**, 869.

LÖNING, T., STAQUET, M.-J., THIVOLET, J. & SEIFERT, G. (1980). Keratin polypeptide distribution in normal and diseased human epidermis and oral mucosa. *Virchows Arch.* (*Pathol. Anat.*) **388**, 273.

LOUIS, C.J., WYLLIE, T.G., CHOU, S.T. & SZTYNDA, T. (1981). Lectin-binding affinities of human epidermal tumours and related conditions *Am. J. Clin. Pathol.*, **75**, 642.

McNUTT, N.S. (1976). Ultrastructural comparison of the interface between epithelium and stroma in basal cell carcinoma and control human skin. *Lab. Invest.*, **35**, 132.

MEROT, Y., DIDIERJEAN, L. & SAURAT, J.-H. (1983). Skin calcium-binding protein immunoreactivity in basal cell carcinoma. *Br. J. Dermatol.*, **109**, 383.

MURPHY, G., FLYNN, T.C., RICE, R.H. & PINKUS, G.S. (1984). Involucrin expression in normal and neoplastic human skin: a marker for keratinocyte differentiation. *J. Invest. Dermatol.*, **82**, 453.

NELSON, D.L., LITTLE, C.D. & BALIAN, G. (1983). Distribution of fibronectin and laminin in basal cell epitheliomas. *J. Invest. Dermatol.*, **80**, 446.

NEMANIC, M.K., FRITSCH, P.O. & ELIAS, P.M. (1982). Perturbations of membrane glycosylation in retinoid-treated epidermis. *J. Am. Acad. Dermatol.*, **6**, 801.

NEMANIC, M.K., WHITEHEAD, J.S. & ELIAS, P.M. (1983). Alterations in membrane sugars during epidermal differentiation: Vizualization with lectins and role of differentiation. *J. Histochem. Cytochem.*, **31**, 887.

NEUBAUER, B.L., CHUNG, L.W.K., McCORMICK, K.A., TAGUCHI, O., THOMPSON, T.C. & CUNHA, G.R. (1983). Epithelial-mesenchymal interactions in prostatic development. II. Biochemical observations of prostatic induction by urogenital sinus mesenchyme in epithelium of the adult rodent urinary bladder. *J. Cell Biol.*, **96**, 1671.

NICOLSON, G.L. (1982). Cancer metastasis. Organ colonization and the cell-surface properties of malignant cells. *Biochim. Biophys. Acta.* **695**, 113.

PINKUS, H. (1979). Factors involved in skin carcinogenesis. *J. Am. Acad. Dermatol.*, **1**, 267.

POLLACK, S.V., GOSLEN, J.B., SHERERTZ, E.G. & JEGASOTHY, B.V. (1982). Biology of basal cell carcinoma. *J. Am. Acad. Dermatol.*, **7**, 569.

ROTHBERG, S. & VAN SCOTT, E.J. (1964). Absence of normal epidermal protein in basal cell tumor. *J. Invest. Dermatol.*, **42**, 141.

SAID, J.W., SASSOON, A.F., SHINTAKU, P. & BANKS-SCHLEGEL, S. (1984). Involucrin in squamous and basal cell carcinomas of the skin: an immunohistochemical study. *J. Invest. Dermatol.*, **82**, 449.

SHIBATA, S., PETERS, P., ROBERTS, D.D., GOLDSTEIN, I.J. & LIOTTA, L.A. (1982). Isolation of laminin by affinity chromatography on immobilised Griffonia simplicifolia lectin. *FEBS Lett*, **142**, 194.

STANLEY, J.R., BECKWITH, J.B., FULLER, R.P. & KATZ, S.I. (1982*a*). A specific antigenic defect of the basement membrane is found in basal cell carcinoma but not in other epidermal tumors. *Cancer*, **50**, 1486.

STANLEY, J.R., HAWLEY-NELSON, P., YAAR, M., MARTIN, G.R. & KATZ, S.I. (1982*b*). Laminin and bullous pemphigoid antigen are distinct basement membrane proteins synthesized by epidermal cells. *J. Invest. Dermatol.*, **78**, 456.

VAN CAUWENBERGE. D., PIERARD, G.E., FOIDART, J.M. & LAPIERE, CH. M. (1983). Immunohistochemical localization of laminin, type IV and type V collagen in basal cell carcinoma. *Br. J. Dermatol.*, **108**, 163.

VAN SCOTT, E.J. & REINERTSON, R.P. (1961). The modulating influence of stromal environment on epithelial cells studied in human autotransplants. *J. Invest. Dermatol.*, **36**, 109.

WEBER, L., KRIEG, T., MULLER, P.K., KIRSCH, E. & TIMPL., R., (1982). Immunofluorescent localization of type IV collagen and laminin in human skin and its application in junctional zone pathology. *Br. J. Dermatol.*, **106**, 267.

WEISS, R.A., GUILLET, G.Y.A., FREEDBERG, I.M., FARMER, E.R., SMALL, E.A., WEISS, M.W. & SUN, T.-T. (1983). The use of monoclonal antibody to keratin in human epidermal disease: alterations in immunohistochemical staining pattern. *J. Invest. Dermatol.*, **81**, 224.